# Performance Evaluation of Montelukast Pediatric Formulations: Part II — a PBPK Modelling Approach

**DOI:** 10.1208/s12248-021-00662-1

**Published:** 2022-01-10

**Authors:** Mariana Guimarães, Maria Vertzoni, Nikoletta Fotaki

**Affiliations:** 1 Department of Pharmacy and Pharmacology, University of Bath, Bath, UK; 2 Department of Pharmacy, National and Kapodistrian University of Athens, Athens, Greece; 3 Centre for Therapeutic Innovation, Department of Pharmacy and Pharmacology, University of Bath, Claverton Down, Bath, BA2 7AY UK

**Keywords:** age-related dissolution, biopharmaceutics, montelukast, oral absorption, pediatrics, physiologically based pharmacokinetic (PBPK) modelling

## Abstract

This study aimed to build a physiologically based pharmacokinetic (PBPK) model coupled with age-appropriate *in vitro* dissolution data to describe drug performance in adults and pediatric patients. Montelukast sodium was chosen as a model drug. Two case studies were investigated: case study 1 focused on the description of formulation performance from adults to children; case study 2 focused on the description of the impact of medicine co-administration with vehicles on drug exposure in infants. The PBPK model for adults and pediatric patients was developed in Simcyp^®^ v18.2 informed by age-appropriate *in vitro* dissolution results obtained in a previous study. Oral administration of montelukast was simulated with the ADAM™ model. For case study 1, the developed PBPK model accurately described montelukast exposure in adults and children populations after the administration of montelukast chewable tablets. Two-stage dissolution testing in simulated fasted gastric to intestinal conditions resulted in the best description of *in vivo* drug performance in adults and children. For case study 2, a good description of *in vivo* drug performance in infants after medicine co-administration with vehicles was achieved by incorporating *in vitro* drug dissolution (under simulated fasted gastric to fed intestinal conditions) into a fed state PBPK model with consideration of the *in vivo* dosing conditions (mixing of formulation with applesauce or formula). The case studies presented demonstrate how a PBPK absorption modelling strategy can facilitate the description of drug performance in the pediatric population to support decision-making and biopharmaceutics understanding during pediatric drug development.

**Graphical abstract:**

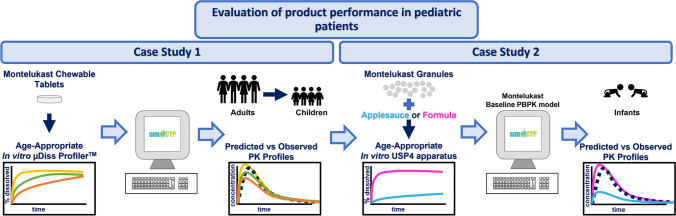

**Supplementary Information:**

The online version contains supplementary material available at 10.1208/s12248-021-00662-1.

## INTRODUCTION

The relevance of physiologically based pharmacokinetic (PBPK) modelling for biopharmaceutic applications in adults is a growing field, as recognised by a recent FDA draft guidance (1). PBPK modelling can be applied in all stages of adult drug development. Many successful reports with the application of oral absorption PBPK modelling with the incorporation of biorelevant dissolution have been reported for adults (2–5).

The introduction of regulatory initiatives and incentives in the United States of America (USA) and Europe has fuelled the development of safe and effective age-appropriate formulations (6–10). The pediatric drug development process should start during the early stages of the adult drug development programme (7). This strategy allows industry sponsors to gather valuable knowledge on adult drug pharmacokinetic (PK) studies, which can later support the description of drug product performance in the pediatric population (11). The development of the pediatric formulation is generally regarded as a more complex, time- and cost-intensive process than the development of an adult formulation (12). Pediatric patients have additional ethical and technical constraints that affect the development of a suitable dosage form; these include the choice of the dosage form and its excipients, palatability, volume of administration, *etc*. (13). Further complications in the pediatric population arise as a result of the lack of formulations designed for the target pediatric population, which lead to drug manipulation practices (14–16), such as tablet splitting and crushing, and/or drug products being mixed with small amounts of food or drink vehicles to facilitate administration (14–16).

The development of age-appropriate biorelevant *in vitro* (solubility and dissolution testing) and *in silico* tools (such as PBPK modelling) is beneficial for the prediction of the *in vivo* performance in pediatric patients. Recently, an integrated *in vivo* and *in silico* approach has also been described for infants (17–19), which focused on the design of *in vivo* adult studies that employ dosing conditions representative of infants. The obtained *in vivo* data was then leveraged to extrapolate drug product performance from adults to infants (17–19). PBPK modelling promotes the mechanistic understanding of formulation performance in pediatric subgroups by allowing incorporation of the knowledge on anatomy and physiology as a function of growth and development (20). The potential applications of pediatric PBPK modelling range from the assessment of safety and efficacy of drugs to the design of clinical trials, extrapolation of adult clinical PK to pediatric patients, and ultimately the possible reduction and or the replacement of clinical trials (6, 8). Fifteen per cent of all the PBPK models submitted for FDA drug approval applications (from 2008 to 2017) have been related to the approval of pediatric medicines (21, 22). Currently, the main intended application of a pediatric PBPK model has been the proposal of initial dosing recommendation for pediatric clinical trials (22). Biorelevant *in vitro* dissolution studies, which take into account pediatric physiology, have started to appear in the literature (13, 23–25). The development of pediatric PBPK models focused on oral absorption coupled with *in vitro* dissolution may lead to discussions around pediatric biopharmaceutics and propose strategies to be used in pediatric drug development for the evaluation of age-related changes on drug product performance (6, 8). Pediatric PBPK absorption modelling examples have started to emerge in the literature (20, 21). Its potential has been highlighted for extrapolating the dissolution safe space, not only in adults but also in pediatric patients (26). PBPK absorption modelling has also been used to support the bridging of drug performance from an immediate release to extended-release formulations of quetiapine in children and adolescents 10 to 17 years of age (27).

This study aims to demonstrate the potential applications of a PBPK absorption modelling strategy coupled with biorelevant *in vitro* dissolution testing for evaluating drug product performance in pediatric patients. Montelukast [a poorly water-soluble compound, clogP = 8.79, pKa _basic_ = 2.8; pKa _acidic_ = 5.7 (28–30)] was chosen as a model drug. Two marketed pediatric formulations are available: Singulair^®^ chewable tablets (indicated for children) and granules (indicated for infants) (31). Montelukast is indicated for the treatment of chronic asthma, and/or exercise-induced asthma, and allergic rhinitis. The PK of montelukast has been studied in adult and pediatric patients (children and infants). In *in vivo* studies in infants, Singulair^®^ granules were mixed with food vehicles to facilitate administration (30, 31). Two case studies were explored that cover different aspects of the evaluation of product performance in pediatric patients. Case study 1 focused on extrapolating formulation performance from adults to children. Case study 2 addressed the impact of medicine co-administration with vehicles on drug exposure in infants. To achieve this, a PBPK model was built in Simcyp^®^ v18.2 informed by age-appropriate *in vitro* dissolution data obtained in a previous study (32).

## MATERIALS AND METHODS

### Pharmacokinetic Data Collection and Data treatment

PK studies of montelukast administered intravenously or orally to adults and pediatric patients were collected from the literature and used to develop and validate the PBPK model. The plasma concentration–time profiles, demographics, and dosing information were extracted from the published reports (33) (Table SI and SII). The observed PK profiles found in the literature were digitalised with WebPlotDigitizer^®^ v4.1 software (33). Two studies reported single-dose administrations of intravenous (IV) infusions of montelukast (3 mg to 18 mg) to adults (34, 35). One study reported montelukast plasma-concentration time profiles after a single oral dose administration of a 50 mg solution, but no demographic information was reported in this study (36). Two clinical datasets, referred to as Study (A) and Study (B), described montelukast PK after administration of oral chewable tablets (4 mg) to fasted adult patients (37). One pediatric study reported IV infusion administration (3.5 mg) to older children and adolescents (6 to 14 years) (35). Regarding oral dosing in pediatric patients, one study reported single-dose administration of montelukast oral chewable tablets (4 mg) to fasted young children (2 to 5 years) (38). Plasma concentration–time profiles of montelukast after administration of single-dose oral granules (4 mg) to different infant sub-groups were reported (36, 39–41). The infants’ prandial state at the time of administration was not reported, but montelukast granules were administered with 5 mL of infant formula or 1 tablespoon of applesauce (formula: 1 to 3 months; applesauce: 3 to 6 months and 6 to 24 months) (36, 39–41). Non-compartmental PK data analysis of observed plasma concentration–time profiles was performed with PKSolver® (add-in program for Microsoft Excel^®^) (42).

### PBPK Model Development: General Structure

**Fig. 1:**
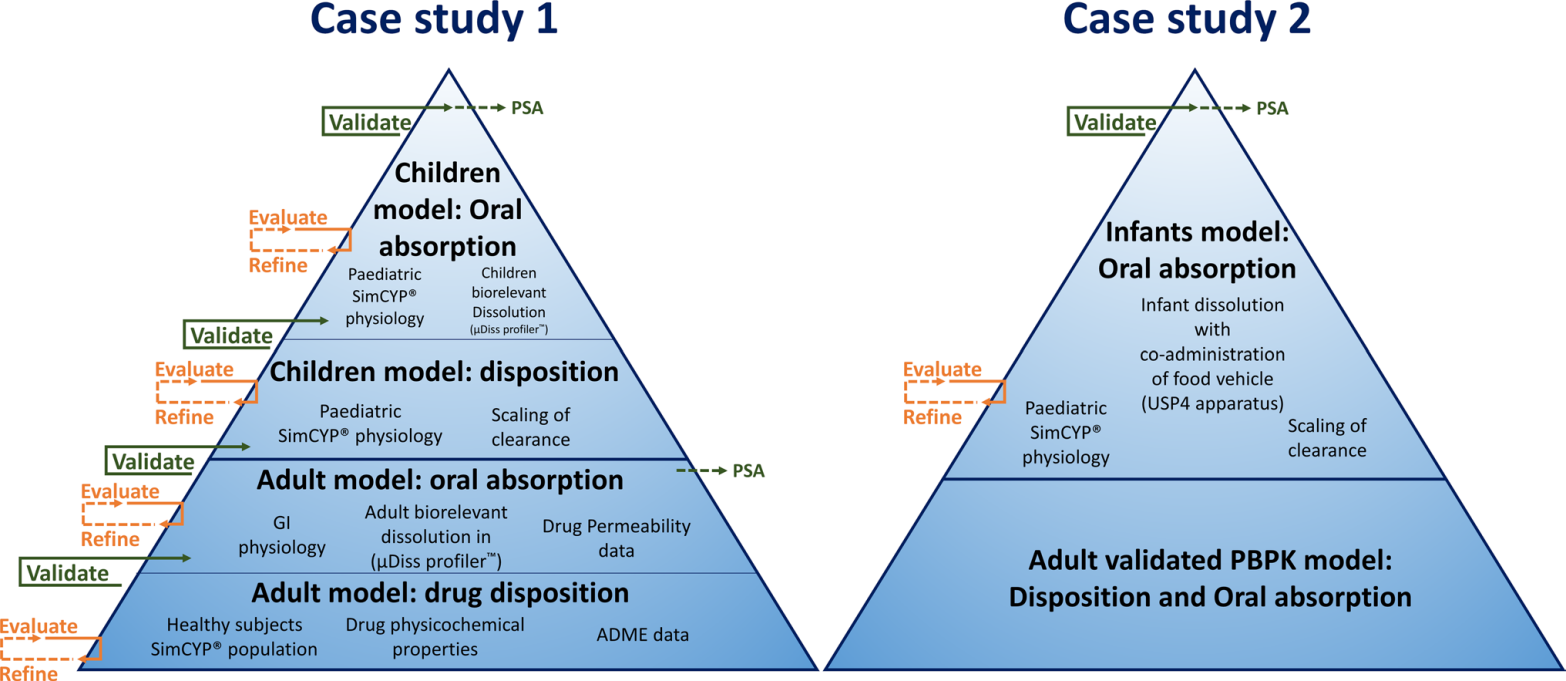
Schematic representation of the workflow describing the physiologically based pharmacokinetic (PBPK) model development of montelukast for the investigated case studies [modified from Guimarães *et al*. (8)]

PBPK modelling and simulations were performed using the Simcyp^®^ software V18.2 (Certara, UK). The PBPK modelling strategy followed the workflow presented in Fig. 1. The PBPK model input parameters that are common to both case studies are summarised in Table I. Montelukast (compound) specific data was obtained from the literature (29, 31, 36).

**Table I:** Summary of Montelukast Input Parameters Used in the Simcyp^®^ Simulator

Input parameter	Value	References/comments
Physicochemical properties and blood binding		
Molecular weight (g/mol)	586.2	(36)
clogP	8.8	(36)
Compound type	Amphoteric	(29)
pKa_1_	5.7	
pKa_2_	2.8	
f_u,p_	0.01	(31)
Blood: plasma ratio	0.62	(36)
Distribution		
Model	Minimal PBPK	
K_in_ (1/h)	0.77	(34)
K_out_ (1/h)	0.99	
V_ss_ (L/Kg)	0.15	
V_sac_ (L/Kg)	0.10	
Elimination		
Cl_IV_ (L/h)	2.85	(34)
Enzyme Kinetics		
CL_int_ (μL/min/pmol) CYP2C8*	3.033	Contribution to metabolic clearance CYP2C8 (72%) CYP3A4 (16%) and CYP2C9 (12%) (44)
CL_int_ (μL/min/pmol) CYP2C9*	0.1569	
CL_int_ (μL/min/pmol) CYP3A4*	0.1015	
Cl_r_ (L/h)	0.0004	(31)
	Absorption	
Model	ADAM™	
P_eff_,_man_ (10^–4^ cm/s)	1.00	Estimated based on Oral solution data (36)
Formulation input	Immediate-release direct input of dissolution as discrete profiles	Details in Table II

^*^ Calculated with the Simcyp^®^ retrograde calculator

The distribution model in all cases was described using a minimal PBPK model with a Single Adjusting Compartment (SAC), a non-physiological compartment that represents a cluster of tissues. The K_in_, K_out_ (first-order rate constants [h^−1^]), V_SAC_ (apparent volume associated with the SAC), and V_ss_ (steady-state volume of distribution) were used. For healthy subjects, the disposition model was developed based according to a 3 mg IV infusion administration of montelukast to adults (34). External validation was performed for IV infusion administration of several dose levels (*i.e.* 9, 7, and 18 mg) (34, 39). For pediatric patients, the distribution SAC parameters [K_in_ (1/h), K_out_ (1/h)] were allometric scaled based on a reference adult bodyweight of 70 kg and an exponent for rate constants is − 0.25 (35, 43).

Montelukast IV clearance was integrated mechanistically using the SimCYP^®^ retrograde calculator to calculate the intrinsic enzymatic clearance (Cl_int_) for each enzyme involved in the metabolic clearance of montelukast. The overall montelukast IV clearance has been reported to be 2.5–3 L/h in adults (34). The relative contribution of metabolic clearance to overall clearance was set as CYP2C8 (72%), CYP3A4 (16%), and CYP2C9 (12%), according to *in vitro* relative activity factor studies (44). The scaling of hepatic metabolic clearance from adults to the pediatric population was automatically applied according to enzyme ontogeny and physiological differences (*i.e.* hepatic blood flow, liver volume, *etc.*) (45). For the pediatric population, the ontogeny of CYP2C8 was applied as described by Upreti and Wahlstrom and validated by Zhou *et al*. (46, 47). Ontogeny of the remaining enzymes was performed with the default software ontogeny functions (48). Following an oral dose of radiolabelled montelukast, 86% of the radioactivity was recovered in 5-day faecal collections, and < 0.2% recovered in urine (31). Renal clearance was scaled to the pediatric population automatically by the software as previously described by Johnson *et al.* (48).

The simulation of oral administration was performed with the Advanced Dissolution Absorption and Metabolism (ADAM™) model. A summary of the input parameters used for montelukast oral simulations in the SimCYP^®^ simulator is presented in Table I. Permeability was estimated based on exposure data after the administration of an oral solution to healthy adult subjects (36).

#### Case Study 1: Extrapolation of Formulation Performance in Children

The formulation input in case study 1 is presented in Table II. Age-appropriate dissolution profiles of Singulair^®^ crushed chewable tablets (4 mg) in adults and children (adjusted dose/volume ratio for each age group) were obtained from the *in vitro* experiments conducted with the µDISS profiler™ (32). Dissolution was inputted in the software as discrete dissolution profiles. Three different dissolution input scenarios were evaluated (Table II):

- Single-stage fasted intestinal profile: Input of the single-stage dissolution profile in fasted state simulated intestinal fluids (FaSSIF-V2) entered as “intestinal profile”. In this scenario, the software takes into account the same dissolution profile for the stomach and intestinal compartments (FaSSIF-V2 dissolution conditions);
- Single-stage fasted gastric and intestinal profiles: Input of the montelukast single-stage dissolution profile in fasted state simulated gastric fluids (FaSSGF) entered as “stomach profile” and single-stage dissolution profile in fasted state simulated intestinal fluids entered as “intestinal profile” (FaSSGF + FaSSIF-V2 dissolution conditions);
- Two-stage fasted gastric to intestinal profile: Input of the two-stage dissolution profile in fasted state simulated gastric fluids (30 min) to fasted state simulated intestinal fluids (30–240 min) entered as “intestinal profile”. In this option, the software considers the same dissolution profile for the stomach and intestinal compartments (FaSSGF to FaSSIF-V2 dissolution conditions).

**Table II:** Summary of Input Parameters Used for Montelukast Oral Administration Simulations in the Simcyp^®^ Simulator. *In vitro* Dissolution Profiles Were Obtained from (32)

Age group (age)	Formulation	Dissolution input		PBPK prandial state
		Scenario	Conditions	
Case-study 1 (µDISS profiler™)				
Adult (> 18 years)	Chewable tablets	Single-stage intestinal profile	FaSSIF-V2	Fasted
		Single-stage gastric and intestinal profile	FaSSGF + FaSSIF-V2	
		Two-stage gastric to intestinal profile	FaSSGF to FaSSIF-V2	
Children (2 to 5 years)		Single-stage intestinal profile	FaSSIF-V2	
		Single-stage gastric and intestinal profile	FaSSGF + FaSSIF-V2	
		Two-stage gastric to intestinal profile	FaSSGF to FaSSIF-V2	
Case-study 2 (USP 4 apparatus)				
Infants (1 to 3 months)	Granules	Two-stage fasted gastric to intestinal profile (drug + formula)	Formula (FaG/FaI)	Fasted
		Two-stage fasted gastric to fed intestinal profile (drug + formula)	Formula (FaG/FeI)	Fed
		Two-stage fasted gastric to intestinal profile (drug + applesauce)	Applesauce (FaG/FaI)	Fasted
Infants (3 to 24 months)				
		Two-stage fasted gastric to fed intestinal profile (drug + applesauce)	Applesauce (FaG/FeI)	Fed

Healthy volunteers Simcyp^®^ population was used in the adult simulations. Adult physiological and anatomical parameters were maintained at the default software values. The Simcyp^®^ pediatric module was used. The pediatric population file gathers information on pediatric demography (age, body height, bodyweight, body surface area) and developmental physiology (*i.e.* liver size, renal function, gastrointestinal size, liver blood flow, *etc.*) as previously described (20, 47). The fasted mean gastric residence time (MGRT) in children was assumed to be the same as adults (*i.e.* 0.27 h).

##### Parameter Sensitivity Analyses

Parameter sensitivity analysis (PSA) was performed for two groups of parameters to understand the impact of the critical variables affecting the PK behaviour of montelukast. The first group of parameters was related to drug properties and included parameters such as % drug dissolved over time (h) and effective permeability (P_eff,man_). Virtual dissolution profiles were constructed to estimate the influence of changes in the formulation’s dissolution characteristics on the PK of montelukast. The % dissolved ranged between 20 and 100% over dissolution times from 0.10 to 4 h. Sensitivity analysis as a function of effective permeability (P_eff,man_) ranged from 0.10 to 2 × 10^−4^ cm/s. The second group of parameters was related to physiologically related parameters, where MGRT was selected due to potential changes between the adult and the pediatric population. MGRT ranged from 0.10 to 2 h. All sensitivity analyses were performed in the fasted state, with a two-stage gastric to intestinal dissolution scenario (FaSSGF to FaSSIF-V2) as input for dissolution.

For the interpretation of the PSA results, simulated PK parameters were compared to the values used in the developed PBPK model (*i.e*. baseline simulation).

#### Case Study 2: Medicine Co-Administration with Food Vehicles in Infants

Description of the formulation input in case study 2 is presented in Table II. The input of montelukast dissolution profiles measured with the USP 4 apparatus simulating infants conditions after montelukast granules co-administration with applesauce or formula (32). Dissolution was inputted in the software as discrete dissolution profiles (Table II). Different dissolution input scenarios were evaluated:

- Two-stage fasted gastric to intestinal profile, drug co-administration with applesauce: Singulair^®^ granules were mixed with 5 mL of applesauce immediately prior to the dissolution test. Dissolution of montelukast granules/applesauce mixture was performed in simulated fasted gastric conditions (0–30 min) to fasted intestinal conditions (30–240 min) (Applesauce + FaG/FaI);
- Two-stage fasted gastric to fed intestinal profile, drug co-administration with applesauce: Singulair^®^ granules were mixed with 5 mL of applesauce immediately prior to the dissolution test. Dissolution of montelukast granules/applesauce mixture was performed in simulated fasted gastric conditions (0–30 min) to fed intestinal conditions (30–240 min) (Applesauce + FaG/FeI);
- Two-stage fasted gastric to intestinal profile, drug co-administration with formula: Singulair^®^ granules were mixed with 5 mL of cow formula immediately prior to the dissolution test. Dissolution of montelukast granules/formula mixture was performed in simulated fasted gastric conditions (0–30 min) to fasted intestinal conditions (30–240 min) (Formula + FaG/FaI);
- Two-stage fasted gastric to intestinal profile, drug co-administration with formula: Singulair^®^ granules were mixed with 5 mL of cow formula immediately prior to the dissolution test. Dissolution of montelukast granules/formula mixture was performed in simulated fasted gastric conditions (0–30 min) to fed intestinal conditions (30–240 min) (Formula + FaG/FeI).

All montelukast dissolution profiles in case study 2 were entered as “intestinal profile”. In this option, the software takes into account the same dissolution profile for the stomach and intestinal compartments.

In the *in vivo* studies available in the literature, 4 mg Singulair^®^ granules were co-administered to different infant sub-groups with formula or applesauce (36, 39–41). The PBPK simulations were set at fasted state for scenarios where dissolution was simulated from a fasted state gastric environment to a fasted state intestinal environment (FaG/FaI). Simulations were performed in the fed state for cases where dissolution was obtained by simulating co-administration with food, in fasted gastric to fed intestinal simulated environment (FaG/FeI) (Table II). The Simcyp^®^ pediatric module was used. Pediatric physiological parameters were maintained at the default Simcyp^®^ simulator values for the infants’ simulations.

##### Parameter Sensitivity Analyses

The parameters investigated in case study 1 (*i.e.* MGRT, P_eff,man_ and dissolution parameters) were also investigated in case study 2 for the various infant subgroups (1 to 2 months; 3 to 6 months and 6 to 24 months). Additionally, a PSA was performed to understand how the maturation of CYP2C8 influences montelukast PK in infants. The CYP2C8 fraction of adult activity ranged from 0.27 to 2.7. The ontogeny of the CYP enzymes can be described with Eq. (1).

1 $$Fraction\; of\; adult={F}_{Birth}+\left(\frac{{(Adult}_{max}-{F}_{Birth)}\times {Age}^{n}}{{Age}_{50}^{n}+{Age}^{n}}\right)$$

where Adult_max_ is the maximal response from adult samples, F_Birth_ is the fraction of adult response at birth, Age_50_ is the age at which half-maximal adult response is obtained, Age is the age of the subject at the time of sample collection in years, and n is an exponential factor. To investigate the effects of changes in CYP2C8 fraction of adult activity on the simulation results, parameters were set as: Adult_max_ ranged from 0.27 to 2.7; F_Birth_ = 1, Age_50_ = 0 and n = 1. Sensitivity analysis was performed for CYP2C8 since it is the main contributing enzyme to the metabolic clearance of montelukast in adults.

All PSA were performed in the fed state with ‘Formula (FaG/FeI)’ dissolution input in infants 1 to 3 months or ‘Applesauce (FaG/FeI)’ dissolution input for sub-groups aged 3 to 6 and 6 to 24 months. For the interpretation of the PSA results, simulated PK parameters were compared to the values used in the developed PBPK model (*i.e*. baseline simulation).

### Trial Design Information

Simulations were performed with 10 trials of 10 subjects. The trial design was performed using the ‘Virtual population’ option in SimCYP^®^, where maximum and minimum age, as well as the proportion of females, was adjusted according to the population of the PK study used for the validation of the PBPK model. Study details are presented in supplementary Table SI and SII.

### PBPK Model Verification

The PBPK model verification was performed by comparison of predicted and observed PK profiles and parameters. The mean predicted plasma concentration–time profiles were assessed by the average fold error (AFE) and validated with the absolute average fold error (AAFE) (Eqs. (2) and (3), respectively) (49).

2 $$\mathrm{AFE}={10}^{\frac{1}{n}\sum \mathrm{log}(\frac{{predicted}_{i}}{{observed}_{i}})}$$

3 $$\mathrm{AAFE}={10}^{\frac{1}{n}\sum \left|\mathrm{log}(\frac{{predicted}_{i}}{{observed}_{i}})\right|}$$

where *n* denotes the number of observed sampling points, *predicted*_*i*_ and *observed*_*i*_ denote the predicted and observed plasma concentration at the sampling time point *i*.

AFE values indicate the trend of the simulated data to underpredict (AFE < 1) or overpredict (AFE > 1) the observed plasma concentration–time profiles, while an AAFE value close to unity represents the precision of the simulations. An AAFE ≤ 2 indicates an acceptable prediction (50).

The relative accuracy of the mean predicted PK parameters describing drug exposure [area under the plasma concentration–time curve (AUC), maximum concentration (C_max_), and time to reach the maximum concentration (T_max_)] was assessed against mean observed PK parameters using the fold error (FE) (Eq. (4)). A FE within a two-fold range (FE values between 0.5 and 2) indicates an acceptable prediction (50).

4 $$\mathrm{FE}=\frac{predicted}{observed}$$

## RESULTS

### Case Study 1

#### Montelukast Adult PBPK Model

**Fig. 2:**
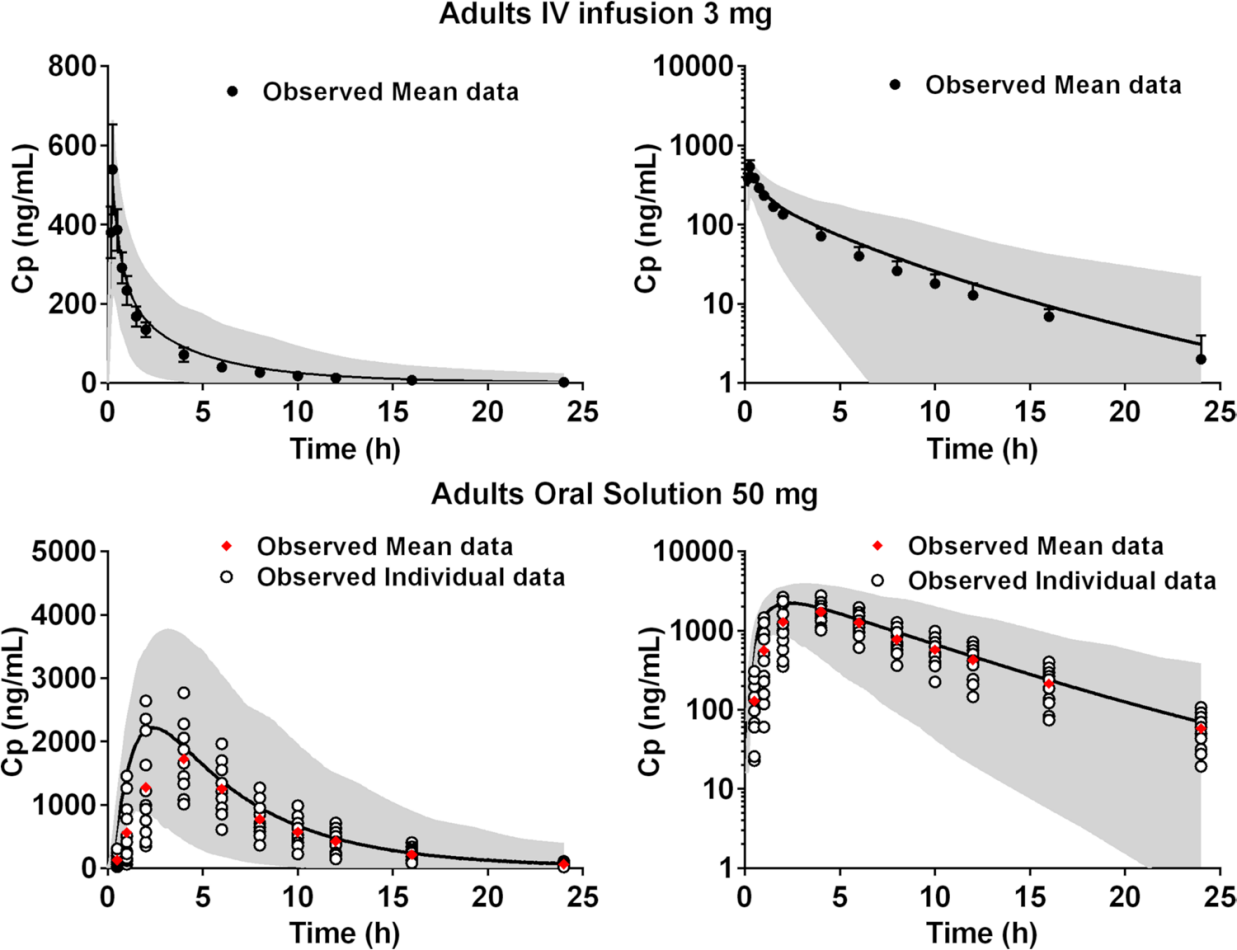
Simulated montelukast plasma concentration–time profiles (solid line, population mean; grey area, 5^th^ and 95^th^ percentile of the population) in adults following by IV administration (3 mg; 15-min infusion) and orally (50 mg oral solution), against observed data in linear (left panels) and log scale (right panels) (34, 36)

**Intravenous administration**: The montelukast PBPK model was verified against the plasma concentration–time profiles obtained after an IV infusion administration (3, 7, 9, and 18 mg). The model simulated PK profile for the IV infusion administration (3 mg) of montelukast is presented in Fig. 2. The PK simulations after IV infusion administration of remaining doses (7, 9, 18 mg) of montelukast are presented in the supplementary materials (Fig. SI). Model verification parameters (FE, AFE, and AAFE) are presented in the supplementary Table SIII. Simulated plasma concentration–time profiles were in good agreement with plasma concentration–time profiles measured after administration of single dose IV infusions (dose range 3 mg to 18 mg). AFE values for all the simulations ranged from 0.9 to 1.2, and AAFE values ranged from 1.1 to 1.2.

**Oral administration**: Effective permeability was estimated with the PBPK model according to the observed plasma concentration–time profiles after the administration of a 50 mg dose of an oral solution of montelukast (by comparison of the simulated with observed plasma concentration–time profile). Drug exposure following administration of an oral solution is independent of drug dissolution; therefore, validation of the model at this stage indicates a good prediction of intestinal permeability. Model verification parameters for the simulation of the administration of montelukast as an oral solution (FE, AFE, and AAFE) are presented in the supplementary Table SIV. Simulation of the administration of montelukast oral solution was in good agreement with the observed plasma concentrations (AAFE = 1.48) and indicated a small overestimation of the mean PK profile (AFE = 1.47) (Fig. 2). In terms of predictability of the PK parameters, T_max_ was slightly underestimated (FE = 0.61) and C_max_ and AUC were both in good agreement with observed data (FE = 1.21 and FE = 1.24, respectively). The permeability value used is in accordance with the values used in previously reported montelukast PBPK models which used P_eff,man_ values between 0.69 and 1.2 × 10^−4^ cm/s (36, 47). Simulated T_max_ was ‘slightly’ earlier than the observed T_max_, but T_max_ FE was still within the two-fold criterion set for model validation.

**Fig. 3:**
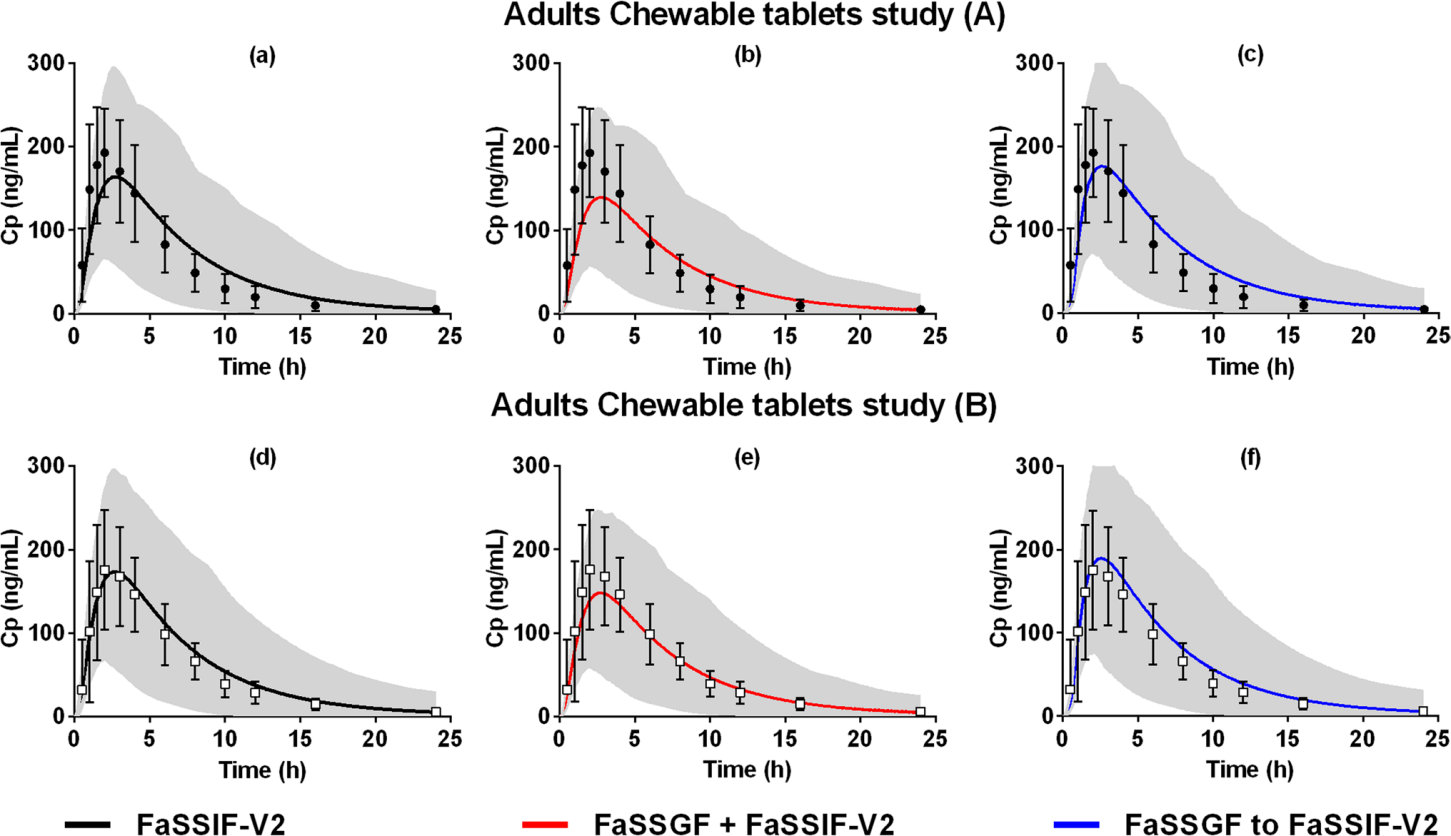
Simulated montelukast plasma concentration–time profiles (solid line, population mean; grey area, 5^th^ and 95^th^ percentile of the population) in adults after administration of montelukast chewable tablets (4 mg) in the fasted state, with different dissolution inputs, against observed data (37); Simulations were (**a**) and (**d**): single-stage fasted intestinal dissolution profiles (FaSSIF-V2), simulations (**b**) and (**e**): single-stage fasted gastric and intestinal profile (FaSSGF + FaSSIF-V2), and simulations (**c**) and (**f**): two-stage fasted gastric to intestinal profile (FaSSGF to FaSSIF-V2)

The description of plasma concentration–time profiles in adults after administration of Singulair^®^ chewable tablets in the fasted state was performed using as model input the biorelevant dissolution profiles measured in the µDISS profiler™ (Table II). The observed and simulated plasma concentration–time profiles after oral administration of Singulair^®^ chewable tablets to adults are presented in Fig. 3. The PBPK model validation and assessment parameters (FE, AFE, and AAFE) are presented in the supplementary materials (Table SIV). Biorelevant dissolution profiles measured with the µDISS profiler™ (dose/volume ratio adjusted for adults) were used as input in the simulations, as described in Table II.

Two clinical datasets, referred to as Study (A) and Study (B), describing montelukast PK after administration of chewable tablets were used for the validation of oral predictions in adults (37). According to the validation parameters (FE, AFE, and AAFE), the PBPK model successfully described the *in vivo* exposure observed in both study designs (Study (A): AAFE 1.42–1.52; Study (B): AAFE 1.13–1.22) (Table SIV). Observed PK parameters from the study (A) and study (B) appear to display similar T_max_ and AUC values, with a lower C_max_ observed for study design (B). The differences between the 2 study designs are related to the minimum age of the subjects (study A = 19 years and study B = 24 years) and the proportion of female subjects (study A = 0.35 and study B = 0.63).

Overall, all simulations of the exposure of montelukast from the Singulair^®^ chewable tablets met the validation criteria (AAFE ≤ 1.52). Both simulations of the administration of montelukast chewable tablets, with dissolution inputs of single-stage fasted intestinal dissolution profile (FaSSIF-V2) (Fig. 3a and d) or two-stage fasted gastric to intestinal dissolution profile (FaSSGF to FaSSIF-V2) (Fig. 3c and f), were in a good agreement with the observed PK data (AAFE range 1.42 to 1.52 in study (A) and 1.13 to 1.22 in study (B)). The best prediction of the mean C_max_ was obtained when the two-stage fasted gastric to fasted intestinal dissolution profile (FaSSGF to FaSSIF-V2) was used as input (FE = 0.92 and 1.08 for study (A) and (B), respectively) (Table SIV). Simulations with single-stage fasted gastric and intestinal dissolution profile as input (FaSSGF + FaSSIF-V2) were in good agreement with observed data (AAFE = 1.46 and 1.16 for study (A) and (B), respectively) with slight underestimation of C_max_ (FE = 0.72 and 0.84 for study (A) and (B) respectively) (Fig. 3b and e).

#### Montelukast Pediatric PBPK Model

**Fig. 4:**
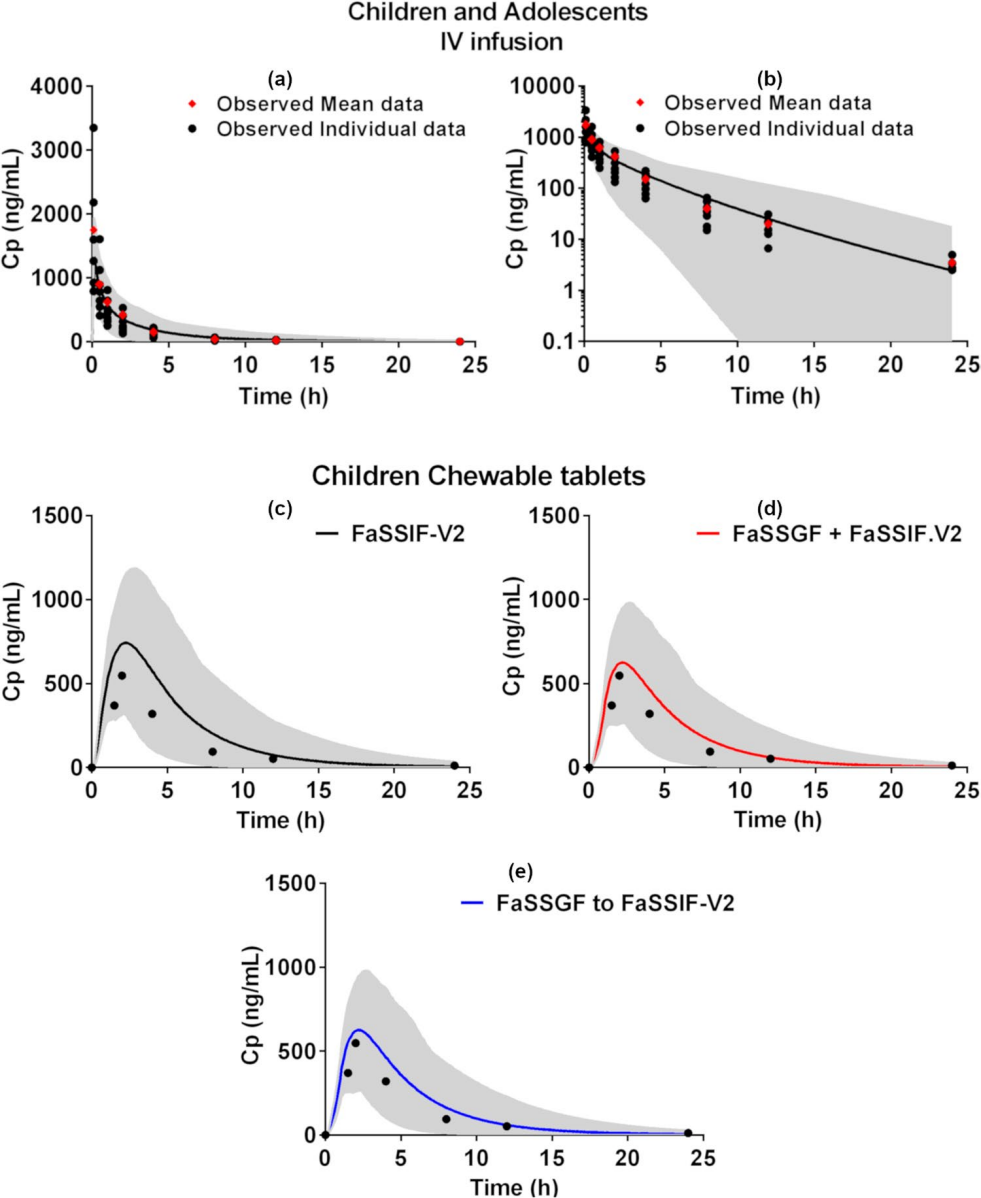
Top-panel: simulated montelukast plasma concentration–time profiles (solid line, population mean; grey area, 5^th^ and 95^th^ percentile of the population) in children and adolescents (6 to 14 years) after IV administration (5-min infusion) of 3.5 mg montelukast against observed data in linear scale (**a**) and log scale (**b**) (35). Bottom-panel: simulated montelukast plasma concentration–time profiles (solid line, population mean; grey area, 5^th^ and 95^th^ percentile of the population) in children (2 to 5 years) after administration of montelukast chewable tablets (4 mg) in the fasted state, with different children dissolution inputs, against observed data (38); simulation (**c**) single-stage fasted intestinal dissolution profile (FaSSIF-V2), simulation (**d**) single-stage fasted gastric and intestinal profile (FaSSGF + FaSSIF-V2), and simulation (**e**) two-stage fasted gastric to intestinal profile (FaSSGF to FaSSIF-V2)

**Children and adolescents intravenous administration:** The simulated plasma concentration–time profiles in children and adolescents after administration of an IV infusion of 3.5 mg of montelukast to children and adolescents (6 to 14 years) are presented in Fig. 4(a,b). The PBPK model validation and assessment parameters (FE, AFE, and AAFE) are presented in the supplementary Table SV. The simulation of IV infusion administration to children and adolescents was in good agreement with the observed plasma concentration–time profiles (AFE = 0.99, AAFE = 1.26).

**Children oral administration:** The simulated plasma concentration–time profiles in children (2 to 5 years) after the oral administration of montelukast Singulair^®^ chewable tablets (4 mg) in the fasted state are presented in Fig. 4(c, d, e). FE, AFE, and AAFE for the performed simulations are presented in the supplementary Table SV. Biorelevant dissolution profiles measured with the µDISS profiler™ (dose/volume ratio adjusted for children) were used as input in the simulations, as described in Table II.

All the predictions for the oral administration of the chewable tablets to children met the validation criteria (AAFE range: 1.52–1.75). Independent of the dissolution profile input, a slight overestimation of the AUC (FE between 1.25 and 1.56) was observed, with the simulated PK parameters being still within the two-fold criterion set for model validation. When using the montelukast single-stage fasted intestinal dissolution profile (FaSSIF-V2) as input, a slight overestimation of simulated C_max_ and AUC was observed (T_max_ FE = 1.15, C_max_ FE = 1.35 and AUC FE = 1.56; Fig. 4c). The inclusion of the gastric dissolution profile with single-stage fasted gastric and intestinal profile (FaSSGF + FaSSIF-V2), or the two-stage fasted gastric to intestinal profile (FaSSGF to FaSSIF-V2) as input to the simulation of oral administration of chewable tablets to children, led to an overall better description of the observed plasma-concentration profile (FaSSGF + FaSSIF-V2, AAFE = 1.59; FaSSGF to FaSSIF-V2, AAFE = 1.52; Fig. 4d, e). The best predictions of the observed PK parameters were obtained when using the two-stage fasted gastric to intestinal dissolution profile as input (T_max_ FE = 1.09, C_max_ = FE 1.14, and AUC FE = 1.25; Fig. 4e).

Following the administration of montelukast chewable tablets to pediatric patients, the number of data points collected during the *in vivo* absorption phase is limited (Fig. 4), which complicates the interpretation of minor mean differences observed in the PBPK modelling when using different dissolution profiles as direct input in the model. This is an inherent limitation commonly associated with modelling PK datasets in special populations.

### Parameter sensitivity analyses

**Fig. 5:**
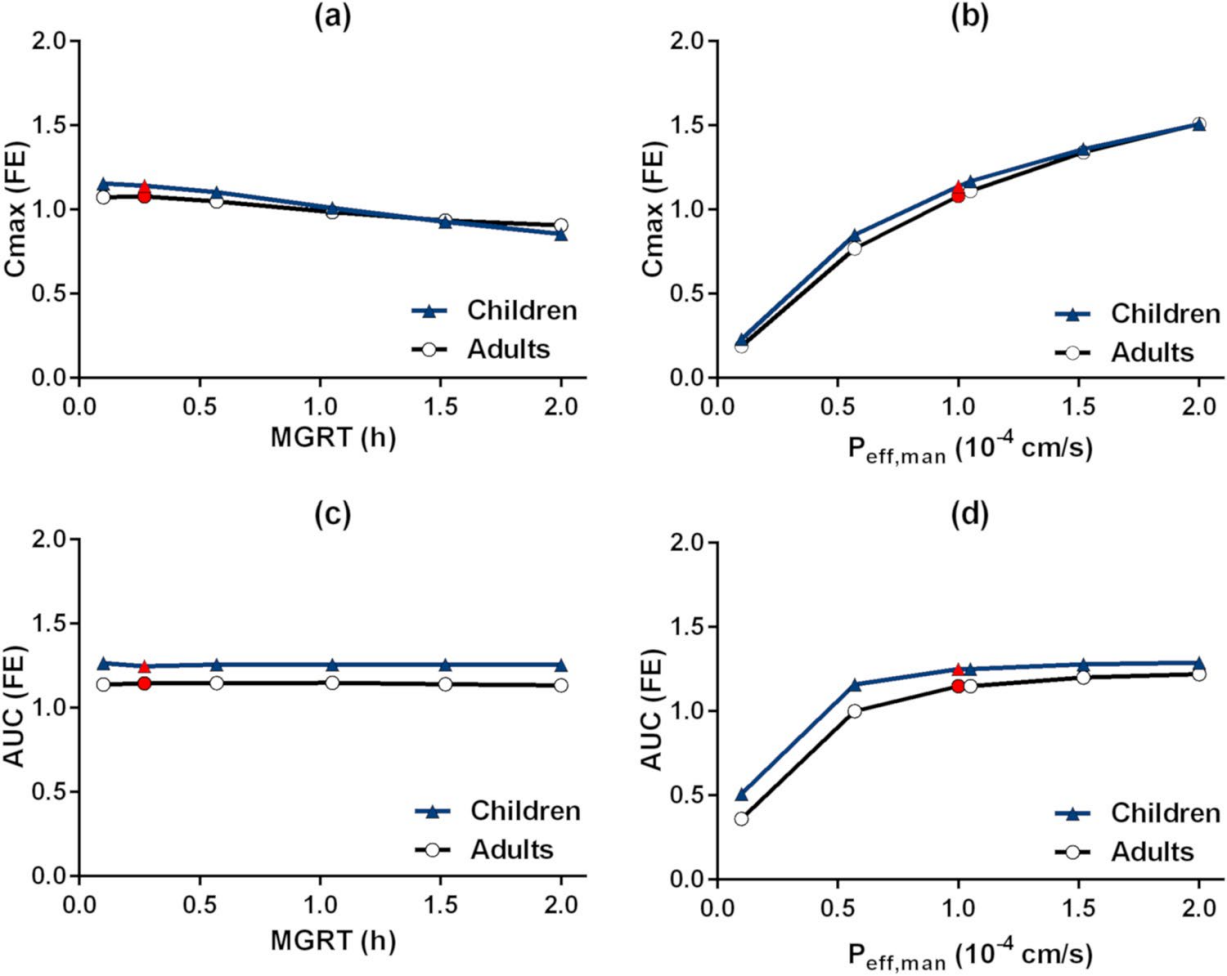
Parameter sensitivity analyses in children and adults for montelukast’s C_max_ and AUC fold error (FE) as a function of mean gastric residence time (MGRT) and effective permeability (P_eff_,_man_); values used in the developed PBPK model (*i.e.* baseline simulation) are shown in red

Results of the PSA conducted to understand the impact of the critical variables affecting the PK of montelukast are presented in Fig. 5. For the interpretation of the PSA, simulation results were compared to the values used in the developed PBPK model (*i.e.* baseline simulation).

The investigated changes in the MGRT (Fig. 5a, c) did not show a substantial impact on the simulated AUC in adults and children. When MGRT increases from 0.27 to 2 h, a small decrease in C_max_ (16% in adults and 23% in children) is observed in comparison to the baseline simulation results. An increase in MGRT from 0.27 to 2 h also leads to prolongation of T_max_ by approximately 46% in adults and by 49% in children, when compared to the baseline simulation results (data not shown).

The results of the sensitivity analysis of montelukast PK parameters as a function of P_eff,man_ are presented in Fig. 5b, d. In adults, a reduction of P_eff,man_ from 1.00 × 10^−4^ cm/s (baseline value) to 0.10 × 10^−4^ cm/s leads to a decrease of C_max_ by approximately 82% (adults) and 79% (children), and a 68% (adults) and 58% (children) decrease in AUC, respectively. On the other hand, an increase in P_eff,man_ from 1.00 × 10^−4^ to 2.00 × 10^−4^ cm/s leads to an increase in C_max_ by 39% and 32%, but only a 7% and 3% increase in AUC, in adults and children respectively.

**Fig. 6:**
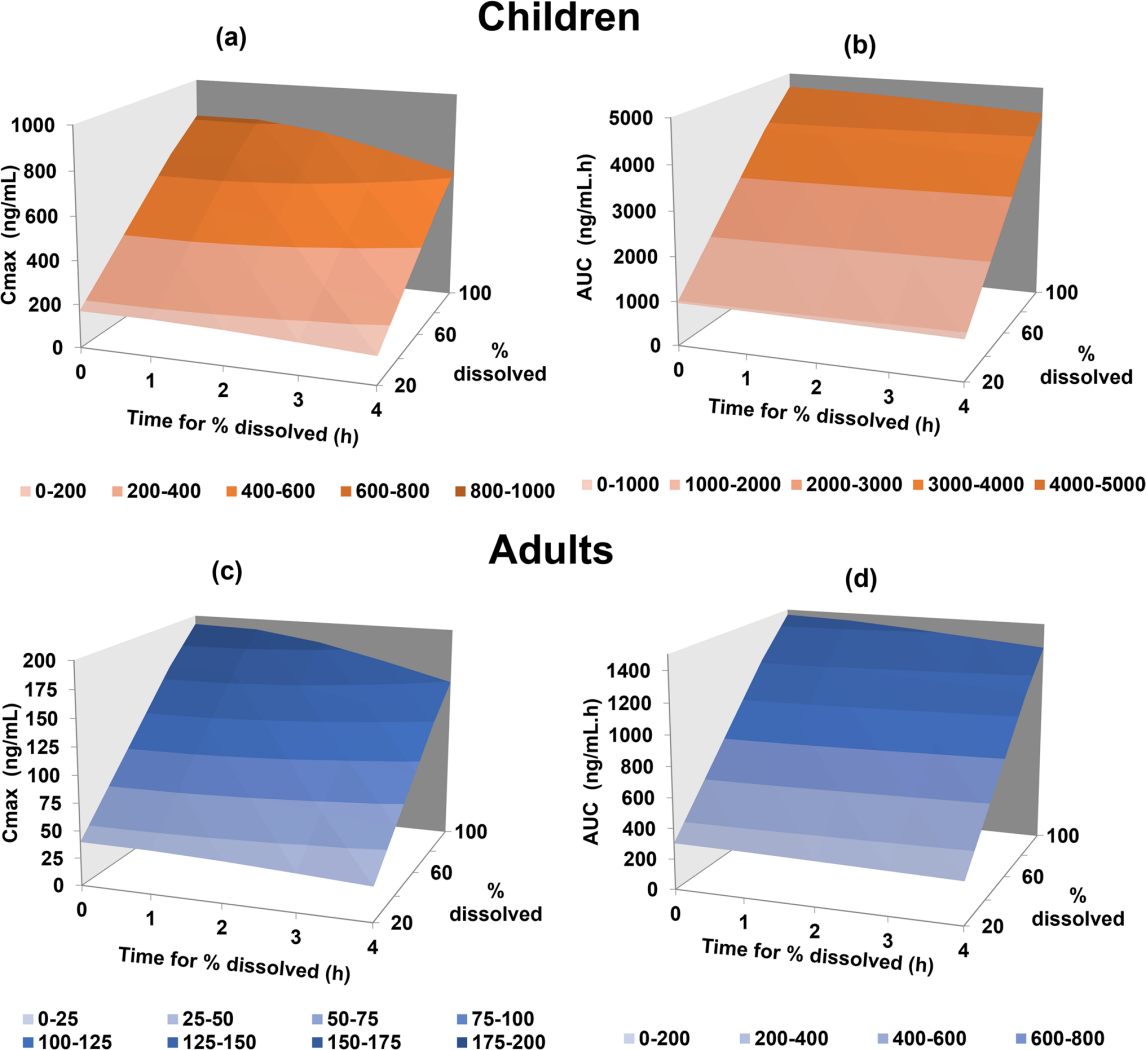
Parameter sensitivity analyses in adults and in children for montelukast’s C_max_ and AUC fold error (FE) as a function of % drug dissolved and time for % dissolved

Sensitivity analysis was also performed on the % dissolved and time of dissolution to understand the impact of these variables on the PK of montelukast (Fig. 6).

To achieve the target plasma concentration–time profile observed in adults, montelukast dissolution would need to reach 80 to 100% dissolved within the first hour after administration (Fig. 6c,d). Slower drug dissolution rates were related to a decrease in C_max_ and notably prolonged T_max_. For example, if 100% of montelukast was dissolved at 4 h in comparison to 0.1 h, C_max_ would decrease by approximately 21% and T_max_ would be prolonged from 2.31 h to 4.58 h (data not shown).

In children, a slightly different *in vivo* drug dissolution behaviour is observed (Fig. 6a,b). To achieve the montelukast target C_max_ and AUC in children, drug dissolution needs to be approximately 60%. If montelukast dissolution was fast and complete (100% dissolved in 0.10 h), children’s C_max_ would be overestimated by 48% and AUC by 61% (in comparison to the observed C_max_ and AUC). If 60% of montelukast chewable tablets are dissolved at 4 h in comparison to 0.10 h, C_max_ would decrease by approximately 24%, AUC by 6% and T_max_ would be prolonged from 1.81 to 4.20 h, overestimating by approximately 110% the observed T_max_ (2 h) (data not shown). Overall, results seem to indicate that montelukast *in vivo* dissolution in children is fast (within the first hour after administration) but does not appear to be complete (contrary to what was observed in adults).

### Case Study 2

#### Infants Oral Administration

**Fig. 7:**
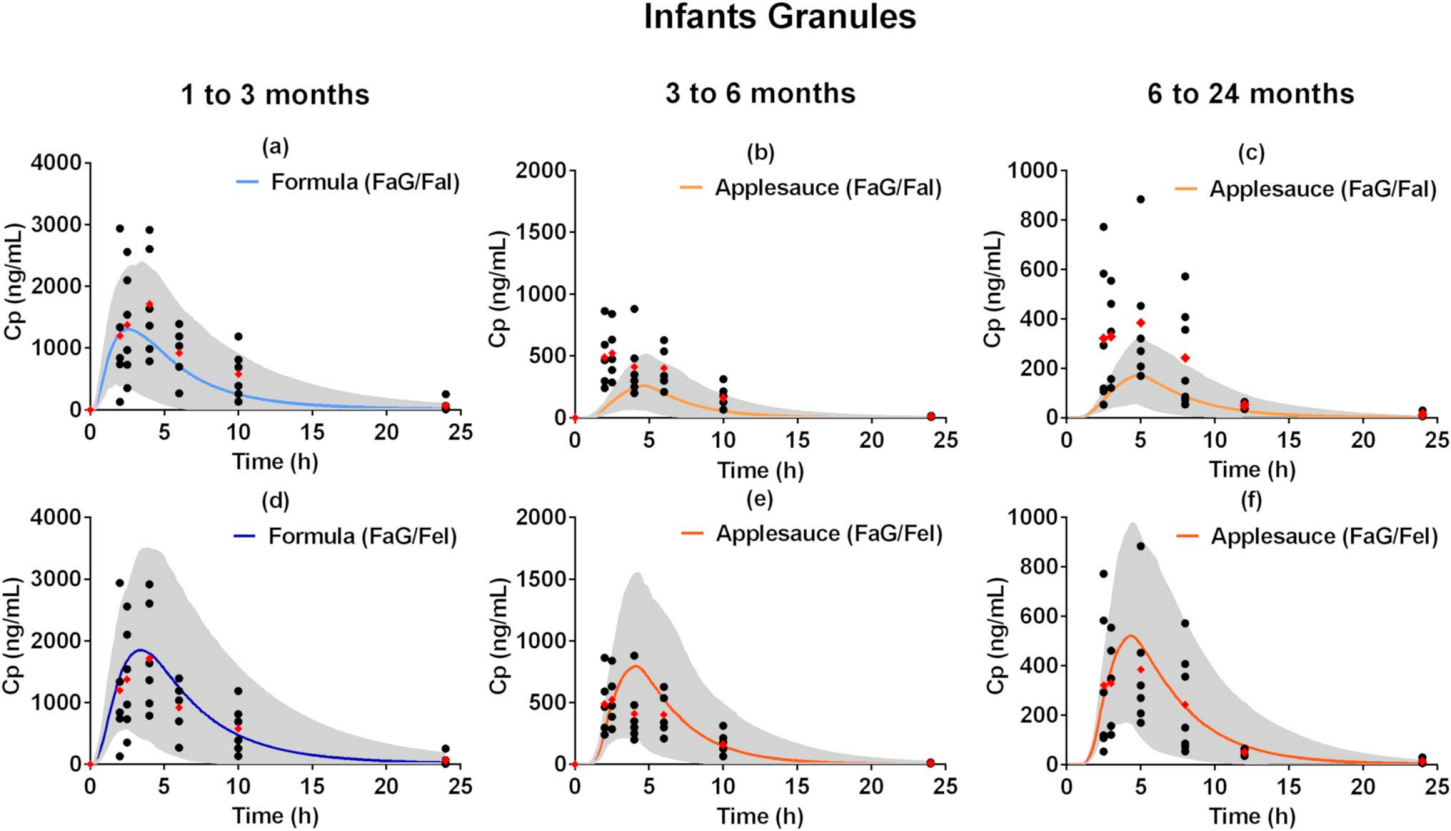
Simulated montelukast plasma concentration–time profiles (solid line, population mean; grey area, 5^th^ and 95^th^ percentile of the population) in infants after administration of a single dose of montelukast granules (4 mg) in the fasted [(**a**)–(**c**)] and fed state [(**d**)–(**f**)], with different dissolution inputs, against observed data (36, 39–41); simulations (**a**) and (**d**): dissolution profiles of montelukast granules co-administered with formula under fasted gastric to fasted intestinal conditions (FaG/FaI) or fasted gastric to fed intestinal, respectively; simulations [(**b**) and (**c**)]: dissolution profiles of montelukast granules co-administered with applesauce under fasted gastric to fasted intestinal (FaG/FaI) or [(**e**) and (**f**)] or fasted gastric to fed intestinal conditions (FaG/FeI)

The simulated plasma concentration–time profiles after oral co-administration of montelukast granules with applesauce or formula to infants are presented in Fig. 7. Model assessment and validation parameters (FE, AFE, and AAFE) for all simulations performed are presented in the supplementary Table SVI. For the fasted state simulations, underestimation of C_max_ and AUC for all age groups was observed, especially for the older infant sub-groups (3 to 6 months and 6 months to 24 months) which received the formulation co-administered with applesauce. Fasted state simulations performed for older infants (aged from 3 to 6 and 6 months to 24 months), did not meet the validation criteria (AAFE ≤ 2) (Fig. 7a,b,c).

For infants aged from 1 to 3 months, a reasonable prediction of the plasma concentration–time profiles was achieved for the fed state simulation with the dissolution input of drug co-administration with formula from fasted gastric to fed intestinal (Formula FaG/FeI; AFE = 0.93, AAFE = 1.37) (Fig. 7d), and all PK parameters were within an acceptable simulation range (FE between 0.90 and 1.12).

For infants aged from 3 to 6 months, predictions of plasma concentration–time profiles were acceptable for the fed state simulation with the dissolution input of drug co-administration with applesauce from fasted gastric to fed intestinal (Applesauce FaG/FeI; AAFE = 1.70) (Fig. 7e). The PK profile was reasonably calculated, with a slight underestimation of later time points (AFE of 0.80 and AAFE 1.70). All simulated PK parameters were within the two-fold validation criterion; a good prediction of AUC was achieved, and a slight overestimation of C_max_ and T_max_ was observed (T_max_ FE = 1.64, C_max_ FE = 1.52, AUC FE = 0.95).

For the infants aged from 6 to 24 months, predictions of plasma concentration–time profiles were acceptable for the fed state simulation with the dissolution input of drug co-administration with applesauce from fasted gastric to fed intestinal (Applesauce FaG/FeI) (Fig. 7f). In this case, AAFE was 1.49 and AFE was 0.89 which indicate a reasonable prediction of the *in vivo* plasma concentration–time profile. The PK parameters were generally well described, with all FE inside the two-fold criterion, and slight overestimation of C_max_ and T_max_ was observed (FE C_max_ = 1.36, and FE AUC = 1.00; T_max_ FE = 1.41). The very slight overestimation of T_max_ in the older infant subgroups (3 to 6 and 6 to 24 months) could be potentially related to the delay observed in the *in vitro* dissolution profile caused by the mixing of the formulation with the applesauce (32); all parameters though were within the validation criteria (two-fold range).

### Parameter sensitivity analyses

**Fig. 8:**
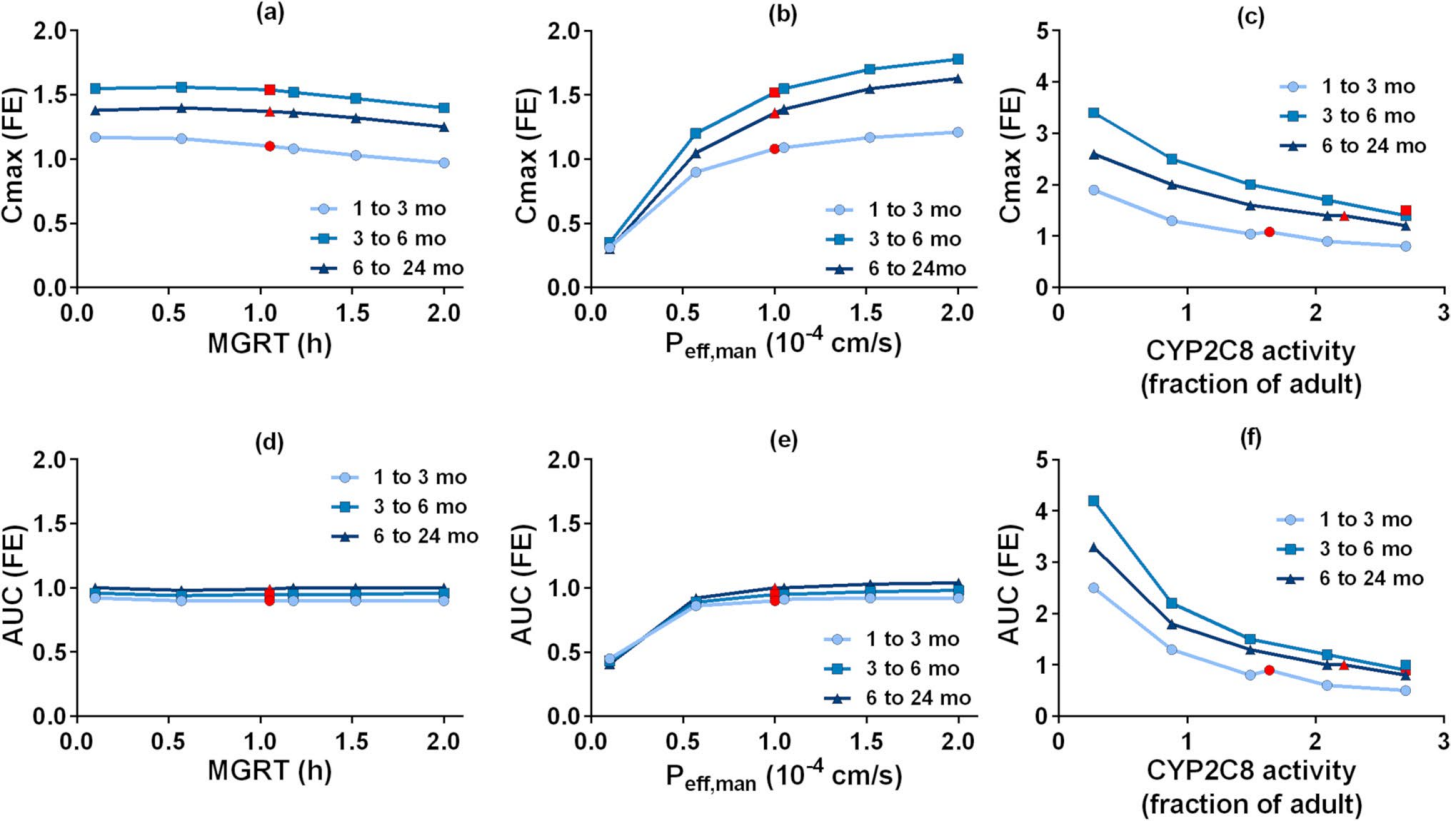
Parameter sensitivity analyses in infants (subgroups: 1 to 3 months, 3 to 6 months, 6 to 24 months) for C_max_ and AUC fold error (FE) as a function of mean gastric residence time (MGRT), effective permeability (P_eff,man_) and CYP2C8 activity (fraction of adult); values used in the PBPK model are shown in red (*i.e.* baseline simulation)

Results from the parameter sensitivity analyses are presented in Fig. 8.

Changes of the MGRT between 0.10 h and 2 h showed a minimal effect in both C_max_ and AUC (less than 11% change compared to the simulation with the value used in the developed PBPK model *i.e.* baseline simulation) (Fig. 8a, d). A decrease in MGRT led to faster T_max_ values in infants in comparison to the baseline simulation (data not shown).

An increase in montelukast P_eff,man_ from 0.10 × 10^−4^ to 2 × 10^−4^ cm/s led to an increase in drug C_max_ in all infant sub-groups (Fig. 8b, e). Increasing the P_eff,man_ to 2.00 × 10^−4^ cm/s resulted in an increase of C_max_ by approximately 12 to 20% and a small increase in AUC (less than 10%) when compared to baseline predictions. A decrease in P_eff,man_ from 1.00 × 10^−4^ to 0.10 × 10^−4^ cm/s was associated with a decrease in C_max_ and AUC (approximately by 75% and 55%, respectively) comparing to the baseline simulation results.

Overall, a decrease in the CYP2C8 fraction of adult activity resulted in an increase in the C_max_ and AUC in infants (Fig. 8c, f). It was also observed that changes in the CYP2C8 fraction of adult activity showed a higher impact on AUC than on C_max_. Changes in CYP2C8 fraction of adult activity showed a higher impact on C_max_ and AUC when compared to the impact of the other tested parameters (*i.e.* MGRT and P_eff,man_). In infants aged from 3 to 6 months, the C_max_ and AUC increased to 124% and 340%, respectively, when the fraction of adult activity was reduced to approximately 0.27 fraction of adult activity, in comparison to the baseline simulation results in this age group.

**Fig. 9:**
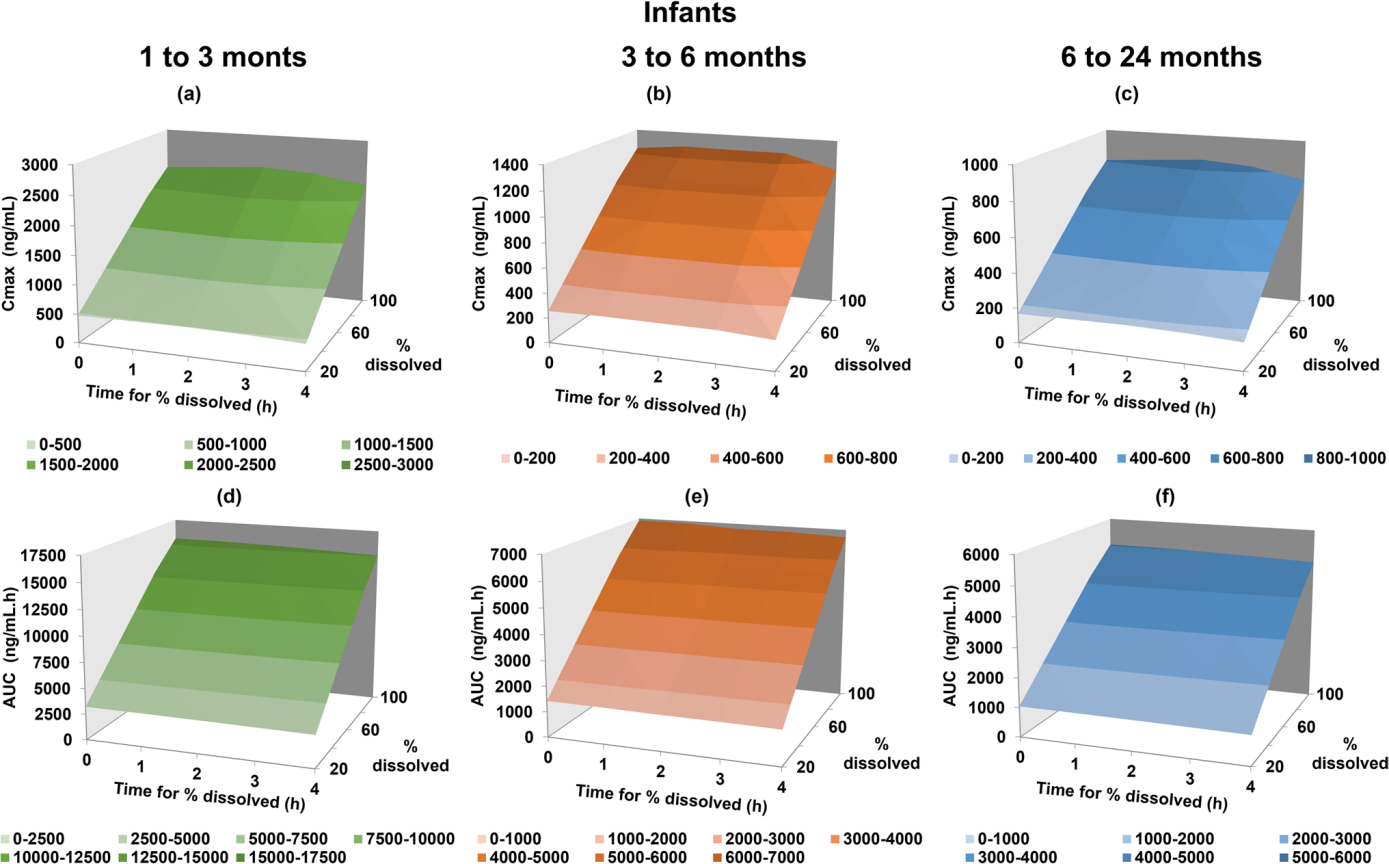
Parameter sensitivity analyses in infants subgroups (1 to 3 months, 3 to 6 months and 6 to 24 months) for C_max_ and AUC as a function of % dissolved and time for % dissolved

In infants between 1 to 3 months, the observed C_max_ was best captured when dissolution extent was between 60 and 80% (Fig. 9a, d). On the other hand, for older infants (age groups 3 to 6 months and 6 to 24 months), the observed C_max_ was best captured if the montelukast dissolution extent was between 40 and 60% (Fig. 9). Slower dissolution rates (*e.g.* 60% dissolved at 4 h) were related to prolonged T_max_ (approximately 80% increase in T_max_, data not shown) and a small decrease in C_max_ and AUC (less than 10% decrease C_max_ and AUC from 0.10 to 4 h), for all infant age groups. Results suggest that the *in vivo* drug dissolution in infants is not complete. Additionally, results of the sensitivity analyses suggest that % drug dissolved *in vivo* in younger infants (1 to 3 months) is slightly higher than in older infants (3 to 24 months).

## DISCUSSION

In this study, the applicability of PBPK modelling for the evaluation of drug product performance in pediatric patients was explored through two case studies. In case study 1, the focus was on the extrapolation of formulation performance from adults to children. Studies were conducted to understand which dissolution input would lead to better prediction of montelukast chewable tablets’ performance between adults and children. Simulations of the administration of Singulair^®^ chewable tablets to adults were all in good agreement with the observed *in vivo* data, and the results suggest that the two-stage dissolution approach was the most successful method in predicting drug performance between adults and children. Estimated fraction absorbed from the chewable tablets was approximately 80% in adults, which is in accordance with the reported *in vivo* bioavailability in this population for the fasted state (31). Simulations in children were also predictive of the *in vivo* data. Estimated montelukast bioavailability from chewable tablets in children was approximately 68%, which is slightly lower than the adult simulated bioavailability. This was further confirmed by the PSA, which showed that the optimal % drug dissolved for the prediction of montelukast PK was lower in children than in adults. The lower dissolution extent is likely related to the higher dose/volume ratio present in children, as revealed by the *in vitro* dissolution studies (32).

In case study 2, the validated PBPK model was used in order to investigate the impact of medicine co-administration with vehicles in infants. Dissolution studies were conducted in fasted gastric to fasted or fed intestinal state and coupled with a respective fasted or fed state PBPK model (as the prandial state of infants was not disclosed in the clinical studies (36, 39–41). Fasted state simulations using *in vitro* fasted state gastric to intestinal dissolution profiles (FaG/FaI conditions) were not predictive of *in vivo* drug exposure, for either of the two vehicles used for drug co-administration. Coupling of *in vitro* dissolution profiles simulating medicine co-administration practices (drug mixed with applesauce/formula in fasted gastric to fed intestinal dissolution conditions) and a fed state PBPK model successfully described the *in vivo* drug performance in infants. The results obtained suggest that simulation of fed state intestinal conditions is more representative of the *in vivo* scenario than fasted state intestinal conditions. These findings are in accordance with the conclusions from Martir *et al*., which showed that infant montelukast PK studies were likely performed in the fed state or that the practice of medicine co-administration with food and drinks might trigger fed state conditions *in vivo* (13). A higher frequency of meals can be seen in pediatric patients, which can lead to the continuous presence of residual food in the stomach and/or intestine, especially for newborns and young infants (8, 51). For the youngest age groups, the food-effect mechanisms are still unknown; therefore, the amounts and/or types of food that can trigger a fed-sate remains undefined (52). Since the definition of fasted and fed states is complex in infants, a food-effect should not be disregarded even where an adult food-effect is not observed (52). The simulated bioavailability in infants was approximately 76% (1 to 3 months — formulation mixed with formula) and 58% (3 to 6 and 6 to 24 months — formulation mixed with applesauce). The higher simulated *in vivo* drug exposure is driven by the higher *in vitro* % montelukast dissolved when mixing the formulation with formula in comparison to mixing the formulation with applesauce. A draft guidance has been issued by the FDA in which the recommended approaches for determining the suitability of the vehicles intended for co-administration of pediatric medicines are discussed (14).

The PBPK absorption modelling approach used in this manuscript addresses the need for tools that help in understanding the impact of medicine co-administration practices on *in vivo* product performance. This approach would be helpful not only to study medicine co-administration with vehicles but also other real-life dosing scenarios of drug manipulation and handling practices. Overall, the continuous optimization of both dissolution and PBPK modelling for pediatric patients will be helpful to support the development of age-appropriate medicines (13, 21).

The described approach can be considered when developing a PBPK model for the investigation of pediatric oral drug absorption. The best data input for a PBPK model will depend on the drug and formulation properties and PK mechanisms involved; therefore, during model development, different *in vitro* strategies should be explored to understand which key mechanisms should be characterized by the model.

A pediatric PBPK model for montelukast has been previously developed which took into consideration experimental *in vitro* measurements of particle size, solubility in fasted simulated gastric and intestinal fluids, and the formulations’ dispersion; this model did not take into account the *in vitro* dissolution performance of montelukast formulations in the different age groups, and it was not clear which ontogeny was used for CYP2C8 (36).

The innovation of our work was to use age-appropriate dissolution testing coupled with a PBPK model. Note that in the current study, the CYP2C8 ontogeny used in infants was partially developed against observed *in vivo* oral data of montelukast by Upreti *et al*. (46). This ontogeny was later externally validated by Zhou *et al*. against oral *in vivo* data of desloratadine which is also metabolised by CYP2C8 (*i.e.* 80% contribution to hepatic metabolism) (47). To improve the relevance of the *in vivo* CYP2C8 ontogeny used, validation should be performed with a higher number of compounds that are highly metabolised by this enzyme. Continuous improvement of pediatric PBPK models and *in vitro* age-related solubility/dissolution methods with high-quality physiological and clinical data is essential and will allow increasing its applicability during pediatric drug development.

## CONCLUSIONS

The developed PBPK model coupled with *in vitro* age-appropriate dissolution tests successfully described the oral exposure of montelukast chewable tablets in adults and children. The calculated bioavailability of montelukast in children was slightly lower than in adults. For poorly water-soluble compounds, to achieve accurate prediction power, the type of dissolution input used needs to be evaluated. The ‘best’ input might not be the same for all drugs and will be certainly related to drugs’ physicochemical properties, drug product characteristics, and study design. In this study, the use of biorelevant two-stage dissolution as input in the PBPK model resulted in the most successful prediction of montelukast performance in adults and children, after administration of montelukast chewable tablets. The developed PBPK model coupled with *in vitro* infant biorelevant dissolution tests simulating *in vivo* dosing conditions successfully described drug exposure in infants after medicine co-administration with food and drinks.

PBPK modelling informed by *in vitro* age-related dissolution tests can be applied to other poorly soluble compounds to evaluate pediatric drug product performance and support pediatric drug development.

## Supplementary Information

Below is the link to the electronic supplementary material.

Supplementary file1 (DOCX 149 KB)
