# Supplementary material for: Performance Evaluation of Montelukast Pediatric Formulations: Part II — a PBPK Modelling Approach
Source: AAPS J. 2022 Jan 10;24(1):27. doi: 10.1208/s12248-021-00662-1 (PMC8816611; doi:10.1208/s12248-021-00662-1)
Supplement: Supplementary file 1 — Supplementary file1 (DOCX 149 KB) [file 12248_2021_662_MOESM1_ESM.docx]

**Supplementary Materials**

**Table SI.** Details of study design after IV administration of montelukast to adults and paediatrics, and overview of observed PK parameters.

| **Age group** | **Age** | **Infusion  time (h)** | **Dose (mg)** | **Pharmacokinetic Parameter** | **Observed** | **Reference** |
| --- | --- | --- | --- | --- | --- | --- |
| **Adults** | 21 to 37 | 0.25 | 3 | T_max_ (h) | 0.25 | (1) |
|  |  |  |  | C_max_ (ng/mL) | 570 |  |
|  |  |  |  | AUC _0-24h_ (ng/mL.h) | 1036 |  |
| **Adults** | 19 to 68 | 0.08 | 7 | T_max_ (h) | 0.08 | (2) |
|  |  |  |  | C_max_ (ng/mL) | 1750 |  |
|  |  |  |  | AUC _0-24h_ (ng/mL.h) | 3256 |  |
| **Adults** | 21 to 37 | 0.25 | 9 | T_max_ (h) | 0.25 | (1) |
|  |  |  |  | C_max_ (ng/mL) | 1922 |  |
|  |  |  |  | AUC _0-24h_ (ng/mL.h) | 3541 |  |
| **Adults*** | 19 to 39 | 0.25 | 9 | T_max_ (h) | 0.25 | (1) |
|  |  |  |  | C_max_ (ng/mL) | 1885 |  |
|  |  |  |  | AUC _0-24h_ (ng/mL.h) | 3280 |  |
| **Adults** | 21 to 37 | 0.25 | 18 | T_max_ (h) | 0.25 | (1) |
|  |  |  |  | C_max_ (ng/mL) | 3639 |  |
|  |  |  |  | AUC _0-24h_ (ng/mL.h) | 7445 |  |
| **Old Children and Adolescents** | 6 to 14 | 0.08 | 3.5 | T_max_ (h) | 0.25 | (2) |
|  |  |  |  | C_max_ (ng/mL) | 3639 |  |
|  |  |  |  | AUC _0-24h_ (ng/mL.h) | 7570 |  |

* all the subjects were females

**Table SII.** Details of study design after oral administration of montelukast to adults and paediatrics, and overview of observed PK parameters.

| **Age group** | **Age** | **Proportion of females** | **Formulation** | **Dose (mg)** | **Prandial State** | **Parameter** | **Observed** | **Reference** |
| --- | --- | --- | --- | --- | --- | --- | --- | --- |
| Adults | NA^a^ | NA^a^ | Solution | 50 | NA | T_max_ (h) | 4.00 | (3) |
|  |  |  |  |  |  | C_max_ (ng/mL) | 1836 |  |
|  |  |  |  |  |  | AUC _0-24h_ (ng/mL.h) | 14560 |  |
| Adults | 19 to 44 years | 0.35 | Chewable Tablets | 4 | Fasted | T_max_ (h) | 2.00 | (4) |
|  |  |  |  |  |  | C_max_ (ng/mL) | 192 |  |
|  |  |  |  |  |  | AUC _0-24h_ (ng/mL.h) | 1183 |  |
| Adults | 24 to 44  years | 0.63 |  |  | Fasted | T_max_ (h) | 2.00 | (4) |
|  |  |  |  |  |  | C_max_ (ng/mL) | 175.7 |  |
|  |  |  |  |  |  | AUC _0-24h_ (ng/mL.h) | 1265 |  |
| Children | 2 to 5  years | 0.53 | Chewable Tablets |  | Fasted | T_max_ (h) | 2.00 | (5) |
|  |  |  |  |  |  | Cmax (ng/mL) | 548 |  |
|  |  |  |  |  |  | AUC _0-24h_ (ng/mL.h) | 2883 |  |
| Infants | 6 to 24  months | 0.59 | Granules |  | NA | T_max_ (h) | 3.00^b^ | (3, 6) |
|  |  |  |  |  |  | C_max_ (ng/mL) | 383 |  |
|  |  |  |  |  |  | AUC _0-24h_ (ng/mL.h) | 3170 |  |
| Infants | 3 to 6  months | 0.36 | Granules |  | NA | T_max_ (h) | 2.50 | (3, 7) |
|  |  |  |  |  |  | C_max_ (ng/mL) | 523 |  |
|  |  |  |  |  |  | AUC _0-24h_ (ng/mL.h) | 4580 |  |
| Infants | 1 to 3 months | 0.42 | Granules |  | NA | T_max_ (h) | 3.00 | (3, 8) |
|  |  |  |  |  |  | C_max_ (ng/mL) | 1715 |  |
|  |  |  |  |  |  | AUC _0-24h_ (ng/mL.h) | 14292 |  |

^a^ demographics and study design were not disclosed, simulations for oral administration of montelukast oral solution were performed in the fasted state assuming an age range from 19 to 44 years; all subjects were males

^b^ median value

**Table SIII.** Observed and predicted PK parameters and calculated FE, AFE, and AAFE after IV infusion administration of montelukast to adults.

| **Dose (mg)** | **PK**  **Parameter** | **Observed**  **value** | **Predicted**  **value** | **FE** | **AFE** | **AAFE** |
| --- | --- | --- | --- | --- | --- | --- |
| 3 | T_max_ (h) | 0.25 | 0.25 | 0.99 | 1.30 | 1.33 |
|  | C_max_ (ng/mL) | 570 | 512 | 0.90 |  |  |
|  | AUC _0-24h_ (ng/mL.h) | 1037 | 1217 | 1.17 |  |  |
| 7 | T_max_ (h) | 0.08 | 0.13 | 1.65 | 0.94 | 1.17 |
|  | C_max_ (ng/mL) | 1750 | 1209 | 0.69 |  |  |
|  | AUC _0-24h_ (ng/mL.h) | 3257 | 2809 | 0.86 |  |  |
| 9 | T_max_ (h) | 0.25 | 0.25 | 0.99 | 1.13 | 1.25 |
|  | C_max_ (ng/mL) | 1923 | 1535 | 0.80 |  |  |
|  | AUC _0-24h_ (ng/mL.h) | 3541 | 3659 | 1.03 |  |  |
| 9* | T_max_ (h) | 0.25 | 0.25 | 1.00 | 1.32 | 1.33 |
|  | C_max_ (ng/mL) | 1886 | 1816 | 0.96 |  |  |
|  | AUC _0-24h_ (ng/mL.h) | 3281 | 4076 | 1.24 |  |  |
| 18 | T_max_ (h) | 0.25 | 0.25 | 0.99 | 1.03 | 1.13 |
|  | C_max_ (ng/mL) | 3639 | 3070 | 0.84 |  |  |
|  | AUC _0-24h_ (ng/mL.h) | 7445 | 7319 | 0.98 |  |  |

* All subjects were females

**Table SIV** Observed and predicted PK parameters and calculated FE, AFE, and AAFE after oral administration of montelukast to adults.

| **Formulation** | **Dose (mg)** | **Dissolution Input** | **PK**  **Parameter** | **Observed**  **value** | **Predicted**  **Value** | **FE** | **AFE** | **AAFE** |
| --- | --- | --- | --- | --- | --- | --- | --- | --- |
| Solution | 50 | not applicable | T_max_ (h) | 4.00 | 2.44 | 0.61 | 1.47 | 1.48 |
|  |  |  | C_max_ (ng/mL) | 1837 | 2215 | 1.21 |  |  |
|  |  |  | AUC _0-24h_ (ng/mL.h) | 14203 | 17580 | 1.24 |  |  |
|  | **Knorr2010 study (A)** | | | | | | | |
| Chewable  Tablets | 4 | Single-Stage: FaSSIF-V2 | T_max_ (h) | 2.00 | 2.66 | 1.33 | 1.07 | 1.42 |
|  |  |  | C_max_ (ng/mL) | 193 | 164 | 0.85 |  |  |
|  |  |  | AUC _0-24h_ (ng/mL.h) | 1184 | 1315 | 1.11 |  |  |
|  |  | Single-Stage: FaSSGF + FaSSIF-V2 | T_max_ (h) | 2.00 | 2.77 | 1.39 | 0.91 | 1.46 |
|  |  |  | C_max_ (ng/mL) | 193 | 139 | 0.72 |  |  |
|  |  |  | AUC _0-24h_ (ng/mL.h) | 1184 | 1126 | 0.95 |  |  |
|  |  | Two-Stage: FaSSGF to FaSSIF-V2 | T_max_ (h) | 2.00 | 2.53 | 1.27 | 1.05 | 1.52 |
|  |  |  | C_max_ (ng/mL) | 193 | 176 | 0.92 |  |  |
|  |  |  | AUC _0-24h_ (ng/mL.h) | 1184 | 1369 | 1.16 |  |  |
|  | **Knorr2010 study (B)** | | | | | | | |
|  | 4 | Single-Stage: FaSSIF-V2 | T_max_ (h) | 2.00 | 2.69 | 1.35 | 1.08 | 1.13 |
|  |  |  | C_max_ (ng/mL) | 176 | 173 | 0.99 |  |  |
|  |  |  | AUC _0-24h_ (ng/mL.h) | 1265 | 1377 | 1.09 |  |  |
|  |  | Single-Stage: FaSSGF + FaSSIF-V2 | T_max_ (h) | 2.00 | 2.66 | 1.33 | 0.92 | 1.16 |
|  |  |  | C_max_ (ng/mL) | 176 | 148 | 0.84 |  |  |
|  |  |  | AUC _0-24h_ (ng/mL.h) | 1265 | 1180 | 0.93 |  |  |
|  |  | Two-Stage: FaSSGF to FaSSIF-V2 | T_max_ (h) | 2.00 | 2.55 | 1.27 | 1.07 | 1.22 |
|  |  |  | C_max_ (ng/mL) | 176 | 190 | 1.08 |  |  |
|  |  |  | AUC _0-24h_ (ng/mL.h) | 1265 | 1449 | 1.15 |  |  |

**Table SV.** Observed and predicted PK parameters and calculated FE, AFE, and AAFE after administration of montelukast to children as an IV infusion or as oral chewable tablets.

| **Age group**  (**years)** | **Administration and Dose (mg)** | **Dissolution Input** | **PK**  **Parameter** | **Observed**  **Value** | **Predicted**  **Value** | **FE** | **AFE** | **AAFE** |
| --- | --- | --- | --- | --- | --- | --- | --- | --- |
| Children and Adolescents  (6 to 14) | IV infusion  3.5 mg | NA | T_max_ (h) | 0.08 | 0.13 | 1.59 | 0.99 | 1.26 |
|  |  |  | C_max_ (ng/mL) | 1840 | 1334 | 0.72 |  |  |
|  |  |  | AUC _0-24h_ (ng/mL.h) | 2209 | 2519 | 1.14 |  |  |
| Children  (2 to 5) | Oral  4 | Single-Stage: FaSSIF-V2 | T_max_ (h) | 2.00 | 2.30 | 1.15 | 1.36 | 1.75 |
|  |  |  | C_max_ (ng/mL) | 548 | 743 | 1.35 |  |  |
|  |  |  | AUC _0-24h_ (ng/mL.h) | 2883 | 4484 | 1.56 |  |  |
|  |  | Single-Stage: FaSSGF + FaSSIF-V2 | T_max_ (h) | 2.00 | 2.30 | 1.14 | 1.19 | 1.59 |
|  |  |  | C_max_ (ng/mL) | 548 | 631 | 1.15 |  |  |
|  |  |  | AUC _0-24h_ (ng/mL.h) | 2883 | 3928 | 1.36 |  |  |
|  |  | Two-Stage: FaSSGF to FaSSIF-V2 | T_max_ (h) | 2.00 | 2.20 | 1.09 | 1.14 | 1.52 |
|  |  |  | C_max_ (ng/mL) | 548 | 626 | 1.14 |  |  |
|  |  |  | AUC _0-24h_ (ng/mL.h) | 2883 | 3594 | 1.25 |  |  |

**Table SVI.** Observed and predicted PK parameters and calculated FE, AFE, and AAFE after oral administration of montelukast granules (4 mg) to infants.

| **Age (months)** | **Dissolution Input** | **PK**  **Parameter** | **Observed**  **Value** | **Predicted**  **Value** | **FE** | **AFE** | **AAFE** |
| --- | --- | --- | --- | --- | --- | --- | --- |
| 6 to 24 | Applesauce (FaG/FaI) | T_max_ (h) | 3.00^a^ | 4.72 | 1.57 | 0.29 | 3.45 |
|  |  | C_max_ (ng/mL) | 383 | 173 | 0.45 |  |  |
|  |  | AUC _0-24h_ (ng/mL.h) | 3171 | 1077 | 0.34 |  |  |
|  | Applesauce (FaG/FeI) | T_max_ (h) | 3.00^a^ | 4.23 | 1.41 | 0.89 | 1.49 |
|  |  | C_max_ (ng/mL) | 383 | 522 | 1.36 |  |  |
|  |  | AUC _0-24h_ (ng/mL.h) | 3171 | 3162 | 1.00 |  |  |
| 3 to 6 | Applesauce (FaG/FaI) | T_max_ (h) | 2.50 | 4.68 | 1.87 | 0.25 | 3.97 |
|  |  | C_max_ (ng/mL) | 523 | 261 | 0.50 |  |  |
|  |  | AUC _0-24h_ (ng/mL.h) | 4581 | 1480 | 0.32 |  |  |
|  | Applesauce (FaG/FeI) | T_max_ (h) | 2.50 | 4.10 | 1.64 | 0.80 | 1.70 |
|  |  | C_max_ (ng/mL) | 523 | 796 | 1.52 |  |  |
|  |  | AUC _0-24h_ (ng/mL.h) | 4581 | 4359 | 0.95 |  |  |
| 1 to 3 | Formula (FaG/FaI) | T_max_ (h) | 3.00 | 2.53 | 0.84 | 0.60 | 1.70 |
|  |  | C_max_ (ng/mL) | 1715 | 1305 | 0.76 |  |  |
|  |  | AUC _0-24h_ (ng/mL.h) | 14293 | 8356 | 0.58 |  |  |
|  | Formula (FaG/FeI) | T_max_ (h) | 3.00 | 3.36 | 1.12 | 0.93 | 1.37 |
|  |  | C_max_ (ng/mL) | 1715 | 1850 | 1.08 |  |  |
|  |  | AUC _0-24h_ (ng/mL.h) | 14293 | 12929 | 0.90 |  |  |

^a^ median value


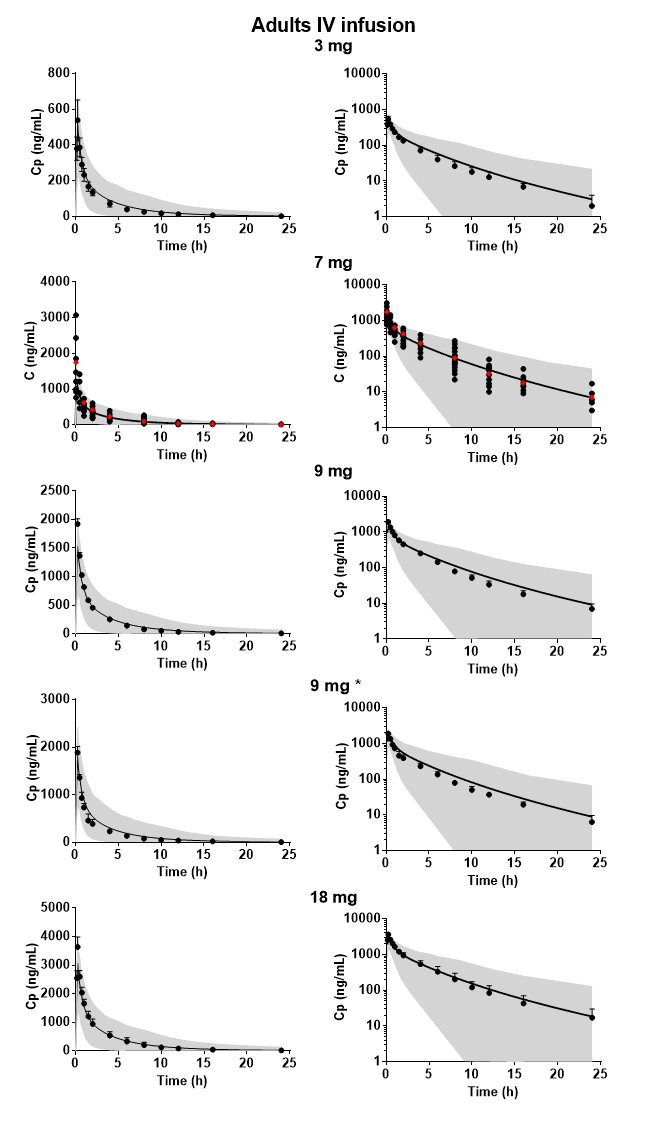


**Figure S1****.** Simulated montelukast plasma concentration-time profiles (solid line, population mean; grey area, 5^th^ and 95^th^ percentile of the population) in adults after IV infusion administration of 3, 7, 9 and 18 mg. * denotes dataset where all subjects were females. Observed data are depicted as filled black circles (1, 2). The mean from the digitalised points is presented as red symbols.

**References**

1. Cheng H, Leff JA, Amin R, Gertz BJ, De Smet M, Noonan N, et al. Pharmacokinetics, bioavailability, and safety of montelukast sodium (MK-0476) in healthy males and females. Pharm Res. 1996;13(3):445-8. doi: 10.1023/a:1016056912698.

2. Ramakrishnan R, Migoya E, Knorr B. A population pharmacokinetic model for montelukast disposition in adults and children. Pharm Res. 2005;22(4):532-40. doi: 10.1007/s11095-005-2493-y.

3. Jones HM, Chen Y, Gibson C, Heimbach T, Parrott N, Peters SA, et al. Physiologically based pharmacokinetic modelling in drug discovery and development: a pharmaceutical industry perspective. Clin Pharmacol Ther. 2015;97(3):247-62. doi: 10.1002/cpt.37.

4. Knorr B, Hartford A, Li XS, Yang AY, Noonan G, Migoya E. Bioequivalence of the 4-mg Oral Granules and Chewable Tablet Formulations of Montelukast. Arch Drug Inf. 2010;3(2):37-43. doi: 10.1111/j.1753-5174.2010.00029.x.

5. Knorr B, Nguyen HH, Kearns GL, Villaran C, Boza ML, Reiss TF, et al. Montelukast dose selection in children ages 2 to 5 years: comparison of population pharmacokinetics between children and adults. J Clin Pharmacol. 2001;41(6):612-9. doi: 10.1177/00912700122010492.

6. Migoya E, Kearns GL, Hartford A, Zhao J, van Adelsberg J, Tozzi CA, et al. Pharmacokinetics of montelukast in asthmatic patients 6 to 24 months old. J Clin Pharmacol. 2004;44(5):487-94. doi: 10.1177/0091270004264970.

7. Knorr B, Maganti L, Ramakrishnan R, Tozzi CA, Migoya E, Kearns G. Pharmacokinetics and safety of montelukast in children aged 3 to 6 months. J Clin Pharmacol. 2006;46(6):620-7. doi: 10.1177/0091270006288324.

8. Kearns GL, Lu S, Maganti L, Li XS, Migoya E, Ahmed T, et al. Pharmacokinetics and safety of montelukast oral granules in children 1 to 3 months of age with bronchiolitis. J Clin Pharmacol. 2008;48(4):502-11. doi: 10.1177/0091270008314251.
